# Widely available, general-purpose generative AI applications for radiology education: determining the teaching quality of synthetic pediatric neuroradiology images

**DOI:** 10.1007/s00247-026-06708-w

**Published:** 2026-07-06

**Authors:** Thomas P. Reith, Donna M. D’Alessandro, Krishnakumari A. Modi, Mohammed Sabawi, Catherina S. Zadeh, Mikhael G. Sebaaly, Lillian M. Lai, Takashi Shawn P. Sato, Simon C. Kao, Yutaka Sato, Michael P. D’Alessandro

**Affiliations:** 1 Department of Radiology, University of Iowa Hospitals and Clinics, Iowa City, IA USA; 2 Department of Radiology, Northwestern University Feinberg School of Medicine, 676 N. St. Clair St., Suite 800, Chicago, IL 60611 USA

**Keywords:** Generative adversarial networks, Generative AI, Neuroradiology

## Abstract

**Background:**

Artificial intelligence (AI) is increasingly incorporated into medical education. Widely available, general-purpose generative AI applications are increasingly capable of producing high-fidelity synthetic medical images, which may have potential educational uses.

**Objective:**

To assess the ability of widely available, general-purpose generative AI applications to produce synthetic pediatric neuroradiology images of teaching quality.

**Materials and methods:**

Ten significant diagnostic errors in pediatric neuroradiologic emergencies were identified and divided into 22 individual clinical scenarios. Two widely available, general-purpose generative AI applications, GPT-5 (OpenAI, San Francisco, CA) and Gemini 3 Pro with the Nano Banana Pro image-generation engine (Google, Mountain View, CA), were prompted to generate five separate images for each scenario, yielding a total of 220 images. Eight fellowship-trained pediatric radiologists independently assessed each image for teaching quality. Ratings were recorded and analyzed. Differences between applications were evaluated using Fisher’s exact test.

**Results:**

Overall, 8.6% of generated images (19/220) were deemed suitable for teaching by more than half of the pediatric radiologists. GPT-5 produced a higher proportion of acceptable images meeting this >50% criterion compared to Gemini 3 Pro (12.7% [14/110] vs. 4.5% [5/110]; *P*=0.031).

**Conclusion:**

At present, widely available, general-purpose generative AI applications should not be used to produce synthetic radiological images for educational purposes. Inaccuracies and inconsistencies limit reliability and may actively impair learning.

**Graphical abstract:**

**Figure:**
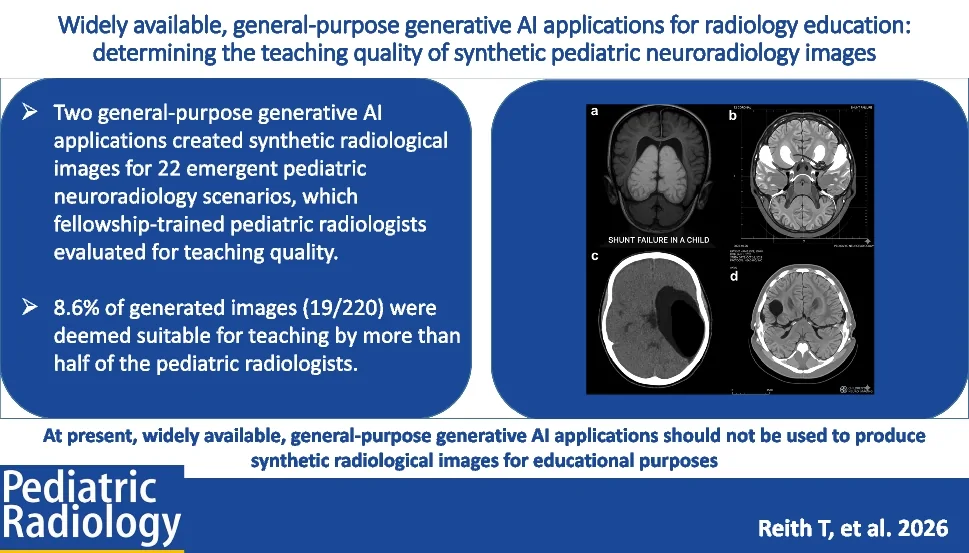

**Supplementary Information:**

The online version contains supplementary material available at https://doi.org/10.1007/s00247-026-06708-w.

## Introduction

Artificial intelligence (AI) has garnered increasing attention in medical education, with much of the discussion focusing on AI literacy and preparing future physicians to use AI systems responsibly in clinical practice [1–4]. Comparatively less attention has been paid to AI’s potential to directly enhance educational content itself. One emerging application is the use of generative AI to create synthetic educational materials, including text, clinical cases, and images. In radiology, where visual pattern recognition is fundamental to training, advances in image-generating AI may create new opportunities to develop customizable teaching cases and supplement limited educational image repositories.

AI has already demonstrated educational value across multiple domains of medical training. Recent applications include AI-powered virtual patient simulators that support diagnostic reasoning [5], surgical simulators that provide adaptive feedback [6], and intelligent tutoring systems that tailor content to individual learners [7]. In radiology, AI-assisted training tools have improved trainee performance in detecting hip fractures [8], rib fractures [9], and pediatric fractures [10], suggesting that AI tools may augment not only clinical practice but also radiology education.

The rapid advancement of image-generating AI models introduces a distinct educational opportunity. Specialized architectures trained specifically on medical images, such as generative adversarial networks (GANs) and diffusion models, can already produce realistic synthetic radiologic images [11, 12]. At the same time, widely available, general-purpose generative AI applications such as GPT and Gemini are becoming increasingly capable of generating high-quality synthetic radiologic images that can be difficult to distinguish from real ones [13]. As these technologies mature, they may enable the creation of realistic, customizable radiologic pathology on demand. Such capability could expand access to rare conditions and allow educators to tailor cases to specific learning objectives or levels of expertise.

Pediatric neuroradiology represents both a compelling application and a stringent test case for generative AI. Trainee exposure to pediatric imaging is often limited by comparatively low case volumes and the subspecialized nature of pediatric practice, especially outside dedicated children’s hospitals. Interpretation requires familiarity with age-dependent developmental anatomy, including evolving myelination patterns, open fontanelles, and variable suture patency, as well as uncommon but clinically significant conditions [14]. Accurately synthesizing pediatric neuroimaging studies thus requires not only realistic depiction of pathology, but also faithful representation of normal developmental anatomy across different ages. Given that missed or delayed diagnoses in children may have lifelong consequences, early and accurate image interpretation is particularly important.

Generative AI offers a potential mechanism to address these challenges by enabling the creation of developmentally appropriate imaging cases spanning a variety of pathologies and age groups. Unlike traditional teaching files, which depend on retrospective case collection (and often institutional case mix), generative systems could allow educators to produce cases prospectively and on demand. In addition, they may enable trainees to generate customized examples tailored to specific learning objectives. For example, on-call residents could potentially use generative systems to create examples of uncommon pathologies or normal developmental variants for reference purposes.

Despite this promise, the ability of widely available, general-purpose generative AI applications to produce teaching-quality radiologic images remains largely unexplored. Prior studies have primarily focused on specialized medical image-generation models, including the creation of synthetic dental panoramic radiographs [15] and CT images of spinal fractures [16]. More recently, widely available, general-purpose generative AI applications have been shown to produce realistic synthetic radiographs [13]. To our knowledge, however, no study has yet tested the capability of widely available, general-purpose generative AI applications to create teaching quality medical images with customizable pathology in pediatric neuroradiology. Therefore, this study assessed the ability of two widely available, general-purpose generative AI applications to produce synthetic pediatric neuroradiology images and evaluated their teaching quality as judged by fellowship-trained pediatric radiologists. The overall intent was to model how a radiology resident, without expertise in prompt engineering, might use a widely available, general-purpose generative AI application as part of routine clinical practice and learning.

## Materials and methods

This study adhered to the Checklist for Artificial Intelligence in Medical Imaging (CLAIM). The three leading widely available, general-purpose generative AI applications were identified. A paid subscription to Google AI Pro was obtained, which provided access to Gemini 3 Pro and its image-generation model, Nano Banana Pro (Google, Mountain View, CA). A subscription to Microsoft Copilot (Microsoft, Redmond, WA), which included access to OpenAI’s GPT-5 model (OpenAI, San Francisco, CA), was provided by the authors’ institution. A third leading widely available, general-purpose generative AI application, Claude (Anthropic, San Francisco, CA), was not capable of native image generation at the time of this study and was therefore not tested.

GPT-5 images were generated between August 21 and September 1, 2025, and Gemini images were generated between November 20 and 23, 2025. All generated images were subsequently reviewed by radiologists during February and March 2026.

Ten most-feared diagnostic errors in pediatric neuroradiologic emergencies were identified from a relevant *RadioGraphics* article [14]. These errors were then divided into 22 individual clinical scenarios designed to illustrate each diagnostic pitfall (Table 1). In selected cases, additional imaging planes were chosen to better demonstrate the error.

**Table 1:** List of ten most-feared diagnostic errors in pediatric neuroradiology and their division into 22 individual scenarios for image generation

	Diagnostic error	Individual clinical scenario used for image generation	Specific modality and plane
1	Detection of early cerebral edema	Early cerebral edema	Head CT without contrast, axial
2	Detection of dural sinus thrombosis	Dural sinus thrombosis	Head CT without contrast, axial
3	Distinction between retropharyngeal edema and early abscess	Retropharyngeal abscess	Neck CT with contrast, axial
		Retropharyngeal edema	Neck CT with contrast, axial
4	Detection of early shunt failure	Early shunt failure	T2-weighted brain MRI without contrast, axial
		Early shunt failure	T2-weighted brain MRI without contrast, coronal
5	Distinction between craniocervical junction fractures and normal developing ossification centers	C1 ossification centers	Spine CT without contrast, axial
		C2 ossification centers	Spine CT without contrast, coronal
		C2 ossification centers	Spine CT without contrast, sagittal
6	Distinction between calvarial fracture and suture	Skull fracture	3D reconstruction
		Skull suture	3D reconstruction
7	Distinction between branchial cleft cyst and retropharyngeal abscess	Infected type II branchial cleft cyst	Neck CT with contrast, axial
8	Time estimation of heterogeneous subdural collections	Acute subdural hematoma	Head CT without contrast, axial
			Head CT without contrast, coronal
		Chronic subdural hematoma	Head CT without contrast, axial
			Head CT without contrast, coronal
9	Distinction between porencephalic cyst and ventriculomegaly	Porencephalic cyst	Head CT without contrast, axial
		Unilateral ventriculomegaly due to an entrapped lateral ventricle	Head CT without contrast, axial
10	Detection of early orbital cellulitis	Early orbital cellulitis	Orbit CT with contrast, axial
			Orbit CT with contrast, coronal
		Cavernous sinus thrombosis	Orbit CT with contrast, axial
			Orbit CT with contrast, coronal

Each AI application (GPT-5 and Gemini) was prompted to generate five separate images for each scenario using the following standardized template:

You are a pediatric neuroradiologist. Create an image of [pathology/normal finding] in a child in the style of [modality] [with/without intravenous contrast] in the [axial/coronal/sagittal] plane.

Bracketed elements were replaced with the appropriate pathology (or normal finding), imaging modality (e.g., CT or MRI), contrast status, and imaging plane for each scenario. For Gemini, Gemini 3 Pro served as the conversational interface, while image generation was performed using the integrated Nano Banana Pro image-generation engine. GPT-5 was accessed through the Microsoft Copilot interface. Default generation settings were used for both applications, and seed and temperature parameters were not modified. Each image was generated in a new chat session to minimize the influence of prior prompts on subsequent outputs, and all images were retained for analysis.

Eight fellowship-trained pediatric radiologists, blinded to the image-generating AI application, independently reviewed the images and corresponding diagnoses in a radiology reading room using 24-inch Dell UltraSharp U2415 liquid-crystal display monitors (Dell Technologies Inc., Round Rock, TX) at a resolution of 1920×1200 pixels. Radiologists were asked to identify “…each image that is an accurate depiction of the disease, such that it is suitable for teaching purposes.” Accordingly, each image was classified by each radiologist as either suitable or not suitable for teaching. For each scenario, between 0 and 5 images could be deemed suitable for teaching by each radiologist. Responses were recorded and subsequently tallied. Differences in overall application accuracy were evaluated using Fisher’s exact test, as were differences in the applications’ ability to generate images in the correct modality and plane. Inter-rater agreement was evaluated using Fleiss’s kappa.

One author was in the room with the radiologists and noted verbal comments during and after they had reviewed images. Another author reviewed each image for correct modality and plane.

Since all images were AI generated and not publicly available, institutional review board approval was not required. GPT-5 was used for language editing and assistance with sentence structure in the drafting and editing of this paper.

## Results

### Overall image quality

A total of 220 images were generated (22 clinical scenarios×5 images×2 AI applications). Overall, 8.6% of images (19/220) were considered to be of suitable quality for teaching by more than half of the reviewing pediatric radiologists (i.e., 5 or more radiologists). Inter-rater agreement across all generated images was moderate (*κ*=0.44).

Image quality differed substantially between applications. For GPT-5, 12.7% of images (14/110) were considered of teaching quality by more than half of the pediatric radiologists, compared with only 4.5% (5/110) for Gemini. Although GPT-5 demonstrated better performance at this >50% threshold, the difference did not reach statistical significance (*P*=0.052). Inter-rater agreement was fair across all GPT-5-generated images (*κ*=0.33) and substantial across all Gemini-generated images (*κ*=0.62).

Using a more stringent agreement threshold, only 6.3% (7/110) of GPT-5 images were rated as teaching quality by more than three-quarters of the radiologists (i.e., 7 or 8 radiologists), while no Gemini images met this >75% agreement threshold (*P*=0.014).

Using the least stringent agreement threshold, 53.6% of GPT-5 images (59/110) were considered of teaching quality by at least one pediatric radiologist, compared with only 10.0% (11/110) of Gemini images (*P*<0.001). Out of all 1,760 individual reviews (220 images×8 reviewing radiologists), 11.9% (209/1760) were considered of teaching quality. 

### Scenario-level performance

**Fig. 1:**
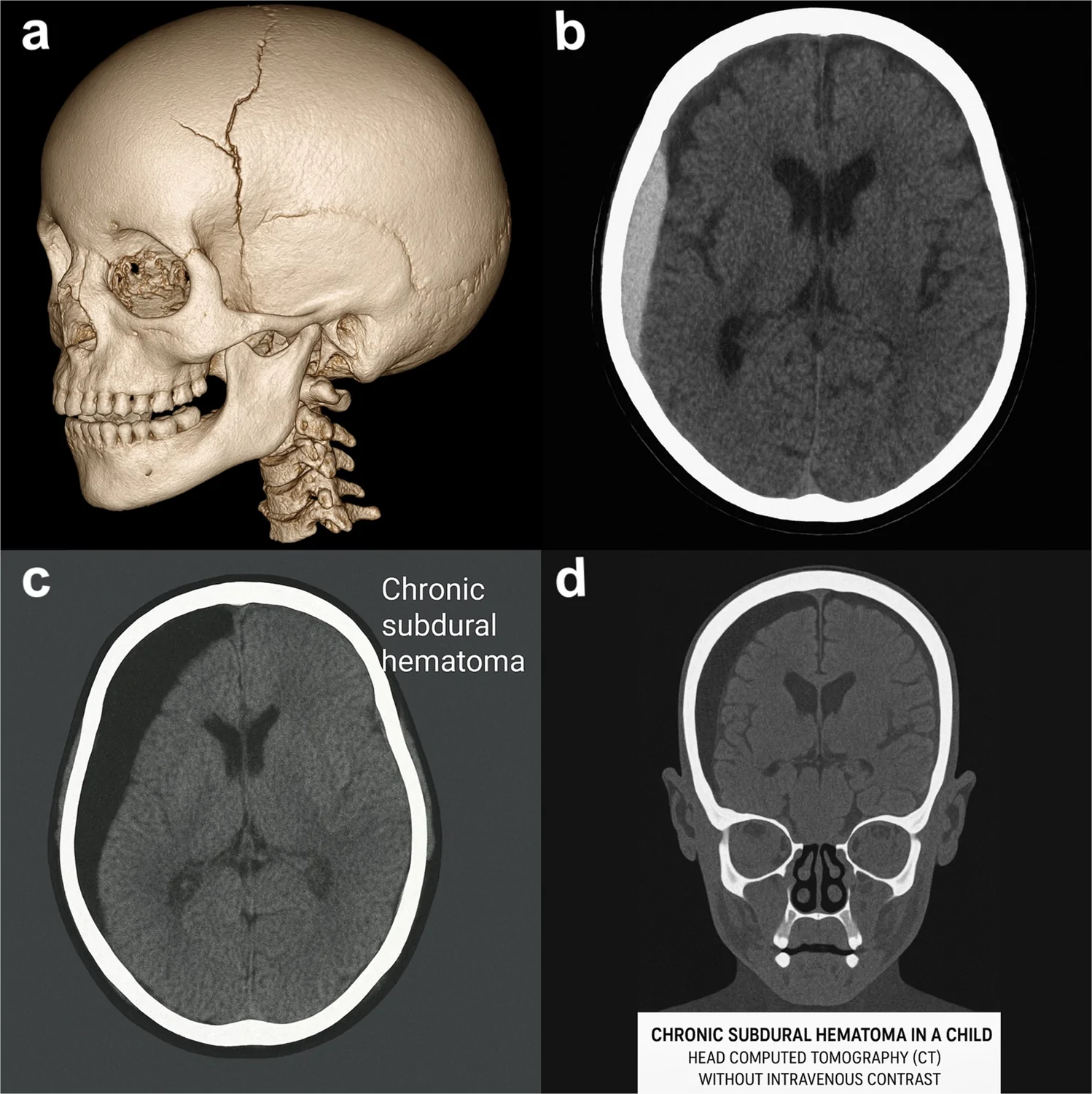
Synthetic three-dimensional skull fracture reconstruction (**a**), axial head CT of an acute subdural hematoma (**b**), axial head CT of a chronic subdural hematoma (**c**), and coronal head CT of a chronic subdural hematoma (**d**) in pediatric patients, all without contrast and generated by GPT-5. A total of 100% (8/8) radiologists rated image (**a**) as being of teaching quality, and 87.5% (7/8) radiologists rated images (**b**), (**c**), and (**d**) as being of teaching quality

**Fig. 2:**
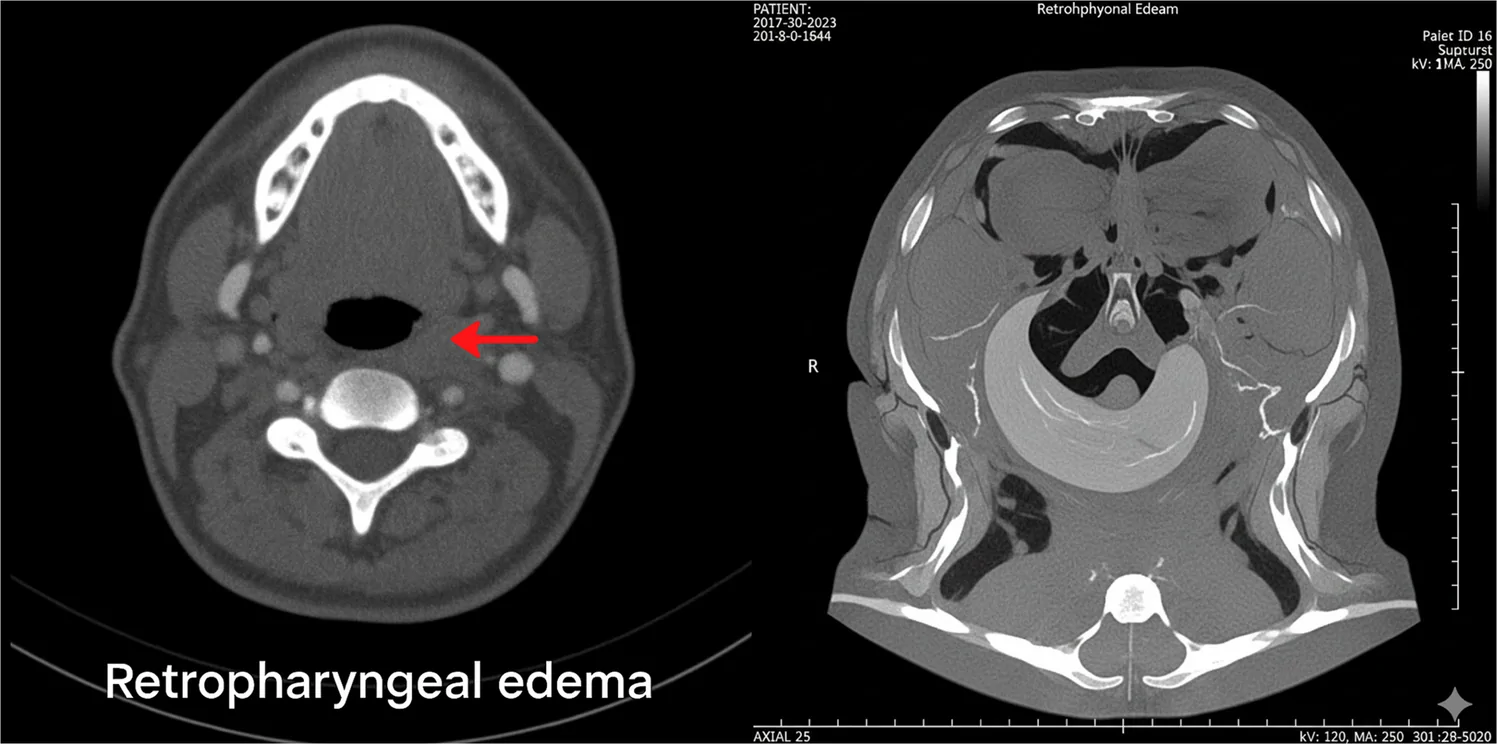
Synthetic axial neck CTs with contrast of retropharyngeal edema in a child generated by GPT-5 (left) and Gemini (right). The GPT-generated image appears more realistic, while the Gemini-generated image exhibits a more “cartoon-like” appearance. One out of 8 radiologists (12.5%) rated the GPT-5 image of teaching quality, and zero radiologists (0%) rated the Gemini image as such

**Fig. 3:**
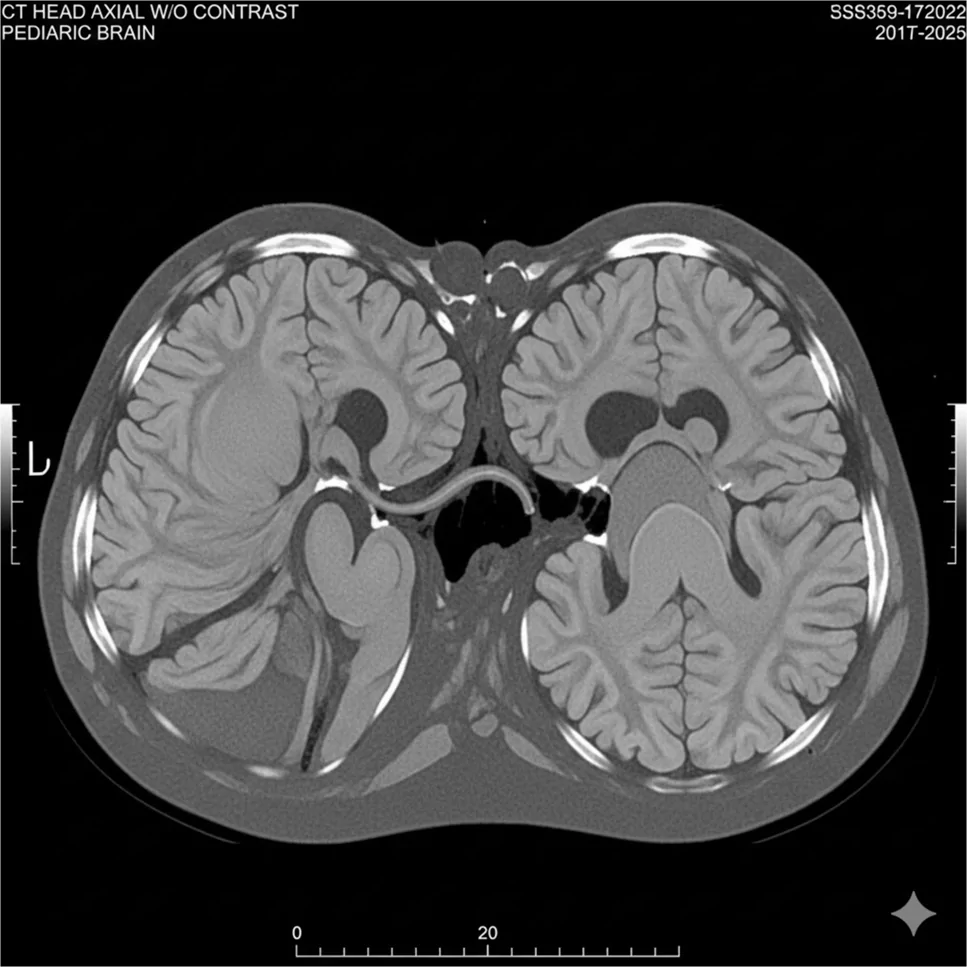
Synthetic axial head CT without contrast of a child with unilateral ventriculomegaly generated by Gemini. Inappropriate anatomical structures are present, including rib-like elements surrounding the cerebral cortex. The cerebral hemispheres appear separated and opposed, resembling kidneys, and a curvilinear structure near the right midline is suggestive of a ureter. Zero radiologists (0%) rated this image as being of teaching quality

**Fig. 4:**
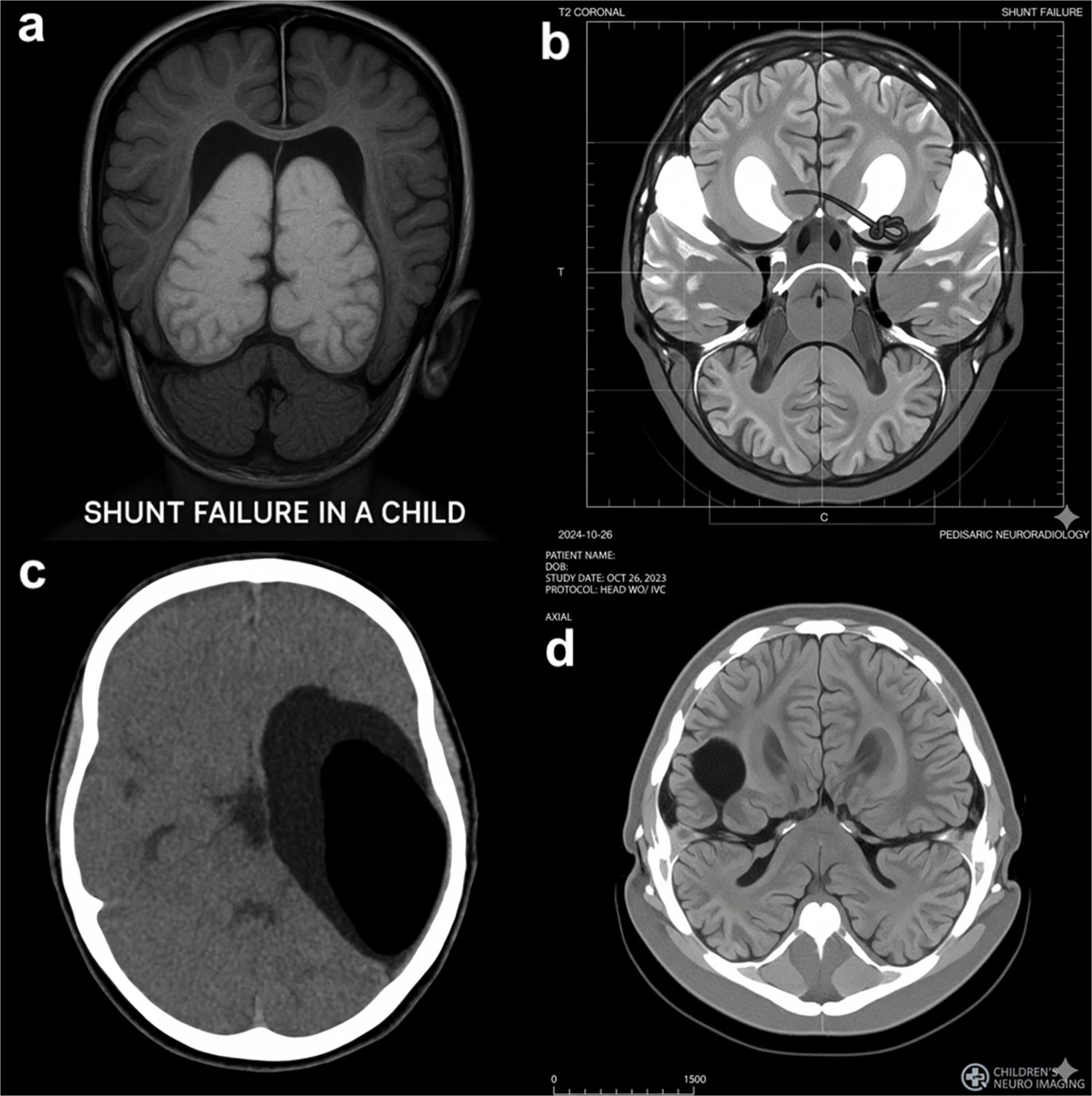
Synthetic T2-weighted coronal brain MRIs without contrast of early shunt failure generated by GPT-5 (**a**) and Gemini (**b**), and axial head CTs without contrast of a porencephalic cyst generated by GPT-5 (**c**) and Gemini (**d**) in pediatric patients. The GPT-5 image in (**a**) demonstrates anatomical inaccuracies, including two additional cerebral lobe-like structures located within the ventricles. The GPT-5 image in (**c**) depicts a cavity containing air surrounded by fluid attenuation, an inaccurate representation of porencephaly. The Gemini images in (**b**) and (**d**) demonstrate osseus rib-like structures surrounding the cerebrum, a recurring anatomical theme observed in Gemini outputs. None of the images (0%) was rated as being of teaching quality by any of the radiologists

At the level of individual scenarios, GPT-5 generated at least one image rated as teaching quality by at least one radiologist in 19 of 22 scenarios (86.4%). Of these, 6 scenarios included at least one image rated as teaching quality by more than half of the radiologists, and 4 scenarios (18.2%) included at least one image meeting the >75% agreement threshold. These high-agreement scenarios involved fractures and intracranial hemorrhages, including three-dimensional skull fracture and suture reconstructions and both acute and chronic subdural hematomas in the axial and coronal planes (see Fig. 1. Note that all annotations on Figs. 1, 2, 3, and 4 were provided unprompted by the AI models).

In contrast, Gemini generated at least one image rated as teaching quality by at least one radiologist in only 4 of 22 scenarios (18.2%). Among these, 2 scenarios (9.1%) included an image meeting the >50% agreement threshold, and no scenario included images meeting the >75% agreement threshold. The high-agreement scenarios involved three-dimensional skull fracture and suture reconstructions.

### Within-scenario consistency

Consistency within individual scenarios was limited for both AI applications. The coefficient of variation ranged from 12–120%, with only 2 scenarios in the average range and all other scenarios having high variability. For GPT-5, only 2 of 22 scenarios had all five generated images rated as teaching quality by at least one radiologist, and only 1 scenario demonstrated unanimous high-level agreement (≥75% of radiologists) across all five images. For Gemini, there was only 1 scenario in which all five generated images were considered of teaching quality by at least one radiologist, and no scenario achieved ≥75% agreement across all images.

GPT-5 more reliably generated images in the correct modality and plane, with 20 of 22 scenarios (90.9%) meeting both criteria across all five images. In contrast, Gemini achieved the correct modality in 2 of 22 scenarios (9.1%) and the correct plane in 7 of 22 scenarios (31.8%). The differences between applications in achieving both correct modality and plane were statistically significant (*P*<0.001). Neither application produced identical images across repeated generations within the same scenario, indicating poor reproducibility.

### Descriptive observations

To the authors, GPT-5’s images were noted to be more realistic, while Gemini’s images exhibited a more stylized or cartoon-like style (Fig. 2). Frequent anatomical inaccuracies in Gemini’s output, such as inappropriate combinations of body structures (e.g., ribcage elements appearing around cranial images) (Figs. 3, 4d), were observed. Although less common, GPT-5 also produced anatomically inaccurate images (Fig. 4).

## Discussion

This study evaluated the ability of two widely available, general-purpose generative AI applications to create synthetic pediatric neuroradiology images of teaching quality. With only 8.6% of images achieving quality consensus among >50% of reviewers, our results demonstrate that the current use of generative AI in this domain is highly variable and, overall, quite poor. Although both applications were able to produce teaching quality outputs in a few select scenarios, overall performance was limited by inconsistent quality, lack of reproducibility, and unreliable depiction of key anatomic and pathologic features.

Nevertheless, the high-quality outputs warrant attention. Figure 1 highlights images from the four scenarios in which GPT-5’s outputs achieved >75% agreement among radiologists. In these instances, the application created near photorealistic images that closely approximated expected imaging characteristics of the underlying pathology, demonstrating the successful replication of visual patterns associated with specific disease processes. Such findings demonstrate the potential of generative AI as a tool for radiology education.

A notable finding is the substantial variability in both image quality and style between the two applications. The reasons for GPT-5’s superior performance are unclear, but may relate to differences in training data, training methodologies, and/or underlying model architecture. The stylistic differences observed may also reflect differences in generative strategies or differing degrees of constraint in the generative process. In particular, the more realistic appearance of GPT-5’s outputs may indicate stronger adherence to learned statistical patterns. In contrast, Gemini’s tendency to combine anatomical structures may indicate greater compositional flexibility and a higher propensity to generate novel features – although often (at this time) anatomically inaccurate ones. These inconsistencies may be related to known spatial reasoning limitations of multimodal large language models [17]. Although such generative flexibility currently limits reliability, it could ultimately prove advantageous if paired with appropriate constraints and validation mechanisms. Nevertheless, the proprietary and largely opaque nature of the widely available, general-purpose generative AI applications used in this study precludes definitive conclusions regarding the factors underlying these performance differences [18].

Prior work on synthetic medical imaging has largely focused on dataset augmentation for training diagnostic algorithms, often using purpose-built models specifically trained on radiologic datasets [19–22]. In contrast, our study evaluated widely available, general-purpose generative AI applications that were not designed or optimized for medical image synthesis and explicitly examined the use of generative AI for educational purposes. This is an important distinction, as features ideal for educational utility – such as reproducibility and anatomical accuracy – differ from those for diagnostic model training. Additionally, the performance of widely available, general-purpose generative AI applications may not be expected to match that of specialized medical image generation systems.

Concerns have also been raised regarding the potential misuse of synthetic images. Tordjman et al. recently demonstrated that AI-generated “deepfake” radiographs were not easily distinguished from authentic images by either human radiologists or AI models themselves [13]. They also noted characteristic image noise or “graininess” in synthetic outputs. Similar artifacts were frequently observed in our dataset, suggesting that this is a persistent artifact of current generative imaging models. Our findings additionally indicate that while contemporary widely available, general-purpose generative AI applications can generate recognizable neuroradiologic concepts, cross-sectional imaging and complex pathology remain challenging to reproduce convincingly.

Our study has implications for the educational use of generative AI. If future developments enable this technology to consistently produce realistic and accurate depictions of pathology, it could become a valuable teaching tool, complementing existing case-based learning resources. For example, it may serve as a reference for on-call residents or non-radiologist physicians, and its use by both clinicians and the general public to generate images is likely to become increasingly common. However, as our findings demonstrate, the current unreliability of generative AI makes it unsuitable for educational use at this time. This concern is particularly important for novice learners, who may lack the expertise required to distinguish authentic findings from fabricated or anatomically inaccurate ones. The use of inaccurate synthetic images in medical education raises ethical concerns, as highly realistic but incorrect depictions may reinforce misconceptions, distort learners’ understanding of normal anatomy, and potentially cause harm. Consequently, educators have an ethical obligation to ensure anatomic accuracy and validate AI systems used for educational purposes against established anatomic references [23].

Limitations of our study include its focus on the narrow domain of pediatric neuroradiology and the relatively small number of generated images. Evaluation of teaching quality is also inherently subjective and may have been influenced by rater experience. Because reviewers were aware that all images were synthetic, the absence of blinding may have introduced bias. Additional potential sources of bias include the use of a single-institution reviewer pool and the lack of comparison with real clinical images. Furthermore, AI-generated outputs are sensitive to prompt phrasing and may reflect biases present in model training data. We employed zero-shot prompting to approximate how radiology residents might interact with widely available, general-purpose generative AI applications in routine educational use; alternative approaches, such as few-shot prompting or more extensive prompt engineering, may have produced different results. Finally, the rapid evolution of generative AI models may substantially improve model performance over time, potentially limiting the generalizability of these findings to future systems. These limitations underscore the need for cautious integration of widely available, general-purpose generative AI applications into radiology (and medical) education and continued evaluation as the technology evolves.

## Conclusion

Currently, widely available, general-purpose generative AI applications should not be used to produce synthetic radiological images for educational purposes. Frequent inaccuracies and inconsistencies substantially undermine reliability and educational value, raising concern that use of this technology in training settings may actively impair learning. Although continued advances in model development may eventually address these limitations, safe and effective integration into radiology education remains premature.

## Supplementary Information

Below is the link to the electronic supplementary material.

ESM 1 (ZIP 183 MB)

## Data Availability

All data supporting the findings of this study, including synthetic images generated, are available within the paper and its Supplementary Information.
